# Risk-Taking and Impulsivity: The Role of Mood States and Interoception

**DOI:** 10.3389/fpsyg.2018.01625

**Published:** 2018-08-29

**Authors:** Aleksandra M. Herman, Hugo D. Critchley, Theodora Duka

**Affiliations:** ^1^Behavioral and Clinical Neuroscience, School of Psychology, University of Sussex, Brighton, United Kingdom; ^2^Sussex Addiction and Intervention Centre, University of Sussex, Brighton, United Kingdom; ^3^Sackler Centre for Consciousness Science, University of Sussex, Brighton, United Kingdom; ^4^Brighton and Sussex Medical School, Brighton, United Kingdom

**Keywords:** UPPS-P, sensation seeking, delay discounting, probability discounting, reflection impulsivity, interoceptive sensibility, emotional state

## Abstract

**Objectives:** The consequences of impulsive decisions and actions represent a major source of concern to the health and well-being of individuals and society. It is, therefore, crucial to understand the factors which contribute to impulsive behaviors. Here, we examined how personality traits of behavioral tendencies, interoceptive sensibility as well as transient mood states predict behavioral performance on impulsivity and risk-taking tasks.

**Method:** 574 (121 males; age 18–45) individuals completed self-report personality measures of impulsivity, reward sensitivity, punishment avoidance as well as interoceptive sensibility, undertook a mood assessment and performed a set of cognitive tasks: delay discounting (temporal impulsivity), probability discounting (risk-taking), and reflection impulsivity task. Data were interrogated using principal component analysis, correlations and regression analyses to test mutual relationships between personality traits, interoceptive sensibility, mood state and impulsive behaviors.

**Results:** We observed a clear separation of measures used, both trait and behavioral. Namely, sensation-seeking, reward sensitivity and probability discounting reflected risk-taking. These were separate from measures associated with impulsivity, both trait (negative and positive urgency, premeditation, perseverance) and behavioral (delayed discounting and reflection impulsivity). This separation was further highlighted by their relationship with the current emotional state: positive affect was associated with increased risk-taking tendencies and risky decision-making, while negative emotions were related to heightened impulsivity measures. Interoceptive sensibility was only associated with negative emotions component.

**Conclusion:** Our findings support the proposal that risk-taking and impulsivity represent distinct constructs that are differentially affected by current mood states. This novel insight enhances our understanding of impulsive behaviors.

## Introduction

Impulsivity describes a set of behaviors characterized by relative dominance of spontaneity over consideration. Examples include a preference toward obtaining immediate gratification over a delayed (yet ultimately more profitable) outcome, making “snap decisions” before evaluating available information, or having difficulty waiting one’s turn, withholding a reaction, or aborting an initiated motor response (Daruna and Barnes, 1993; Moeller et al., 2001). Although, spontaneous actions may be adaptive, for example when the matter is of little importance or when there is little time to make a decision (Dickman, 1990), high levels of impulsivity often result in negative consequences. Correspondingly, impulsivity is associated with poor academic achievement and impaired psychometric performance on reasoning tasks (Schweizer, 2002; Lozano et al., 2014). A high degree of impulsivity is also related to risky driving (Pearson et al., 2013), violent behavior when under the influence of alcohol (Klimkiewicz et al., 2014), diminished self-control and an increased food intake (Guerrieri et al., 2007a,b; Meule and Kübler, 2014), especially while experiencing negative emotions (Van Blyderveen et al., 2016). The importance of impulsivity is increasingly recognized in a clinical setting: Many neuropsychiatric conditions, including addiction, bipolar disorder, and Attention-Deficit Hyperactivity Disorder are characterized by elevated impulsivity (American Psychiatric Association, 2013). Risk-taking is also closely related to impulsivity and predicts the initiation of drug and alcohol use and the pursuit of other hazardous behaviors (e.g., unprotected sex) (Donohew et al., 2000; Ríos-Bedoya et al., 2008).

Impulsivity may determine the integrity of our health and how everyday life flows or falters. It is, therefore, crucial to understand the factors that underlie impulsive behavior and its expression. Moreover, impulsivity is a multidimensional construct (Whiteside and Lynam, 2001; Caswell et al., 2015; Herman et al., 2018), so it is also vital to investigate what factors might differentially influence distinct impulsivity subtypes. Ultimately, improved understanding of modulators of impulsive behavior can enable us to develop better-coping strategies to help impulsive individuals and promote more advantageous decision-making in everyday life. Finally, impulsivity research to date focuses either on university students or certain target populations, e.g., substance abusers or binge drinkers. Hence broad information about the general population is lacking, yet much needed.

One likely modulator of impulsive behavior is affective state (for discussion see Herman et al., 2018). Indeed, people show diminished impulse control (i.e., behave more impulsively) when experiencing negative affect (NA) (Tice et al., 2001). However, it is unknown if subtypes of impulsivity are equally affected by emotional states or whether impulsive behavior is particularly sensitive to specific emotions. Moreover, the role of characterological features contributing to “behavioral style,” for example, personality traits or sensitivity to internal bodily signals (interoception), is not to be underestimated, as these may shape how impulsively individuals respond while experiencing various mood states.

Implicitly one would assume that a measure of trait impulsivity would reflect the degree to which an individual behaves impulsively. However, typically very weak relationships are observed between various trait impulsivity (questionnaire) measures and objective performance on impulsivity tasks (Franken et al., 2008; Cyders and Coskunpinar, 2011; Shen et al., 2014; Caswell et al., 2015). Possibly, interoceptive ability, enabling more accurate detection of internal bodily sensations, e.g., heart rate (Craig, 2009), may determine why and when we behave impulsively. Physiological cues may guide behavior particularly when a potential risk is involved (Damasio, 1996; Bechara et al., 1997; Katkin et al., 2001). For example, in a classic study by Bechara et al. (1997), healthy individuals playing a gambling task generated anticipatory skin conductance responses whenever they considered a choice that turned out to be risky, before they developed an explicit knowledge that the choice was risky. In addition, more recently, good interoceptive ability was found to be associated with more advantageous choices in the Iowa Gambling Task (Werner et al., 2009) and predicted profitable decisions in London financial traders (Kandasamy et al., 2016). Since disadvantageous decision-making is considered a part of impulsivity construct (Winstanley, 2011; Herman et al., 2018), this evidence could suggest that more impulsive individuals may lack interoceptive sensitivity. Alternatively, since highly impulsive individuals appear also to have lower resting levels of arousal compared to peers (Fowles, 2000; Mathias and Stanford, 2003; Puttonen et al., 2008; Schmidt et al., 2013), and engagement in impulsive or risky actions may be a maladaptive way of reaching an “optimal” level of arousal (Zuckerman, 1969; Barratt, 1985; Eysenck and Eysenck, 1985), impulsive individuals may have normal interoceptive sensitivity to changes in their internal state, yet engage in impulsive actions as a means of regulating their arousal level.

Within the current study, we sought to examine the relationship between personality traits of impulsive tendencies, reward sensitivity and punishment avoidance, subjective interoceptive traits (interoceptive sensibility; Garfinkel et al., 2015), current emotional states with behavioral impulsivity. In particular, we were interested which of these variables would be the best predictor of task performance. The UPPS-P impulsive behavior scale (Cyders and Smith, 2007; Whiteside and Lynam, 2001) was used to assess aspects of impulsive tendencies. This scale was selected as it incorporates several dimensions of impulsivity based on personality measures with addition of tendencies for impulsive behaviors while experiencing strong emotions (urgency subscales). Additionally, the Behavioral Inhibition System/Behavioral Activation System Questionnaire (Carver and White, 1994) was employed as a measure of reward sensitivity and punishment avoidance. The Body Perception Questionnaire (Porges, 1993) was used to score general subjective sensitivity to bodily processes (interoceptive sensibility; Garfinkel et al., 2015). The Positive Affect/Negative Affect Scale (PANAS) (Watson et al., 1988) and the Depression, Anxiety, Stress Scale (Henry and Crawford, 2005) were used to assess self-reported emotional state. Risk-taking behavior was assessed from performance on a probability discounting task (PD) (Madden et al., 2009). Distinct facets of impulsive behavior were measured with the Monetary Choice Questionnaire (MCQ) (Kirby et al., 1999), which assesses the ability to delay gratification (temporal impulsivity), and performance of the Matching Familiar Figures Task (MFFT) (Cairns and Cammock, 1978), which measures the degree of information seeking before making a decision (reflection impulsivity).

Since impulsivity is a term which encompasses a wide range of behaviors (Herman et al., 2018), we hypothesized that distinct behavioral dimensions would be predicted by distinct factors. First, as interoception is linked to risk-taking and advantageous decision-making (Werner et al., 2009; Kandasamy et al., 2016), we predicted that individual differences in interoceptive sensibility would predict risk-taking. Second, extending earlier observations (Tice et al., 2001), we predicted that negative emotional states compromise self-control, and thus increase behavioral impulsivity. Third, we predicted that components of the UPPS-P scale, which include emotion-based impulsivity components, would predict objective aspects of behavioral impulsivity.

To test our hypotheses, we conducted an online survey study of participants extending into the general population, providing a more demographically representative sample of the United Kingdom population than earlier studies. Participants completed self-report personality questionnaires, state-mood assessment and interoceptive sensibility questionnaires, and performed specific behavioral tasks to obtain an objective measure of impulsivity and risk-taking.

## Materials and Methods

The study was approved by the University of Sussex Ethical board. Volunteers had to be at least 18 years old to participate. The study was conducted online via Qualtrics platform^1^ between May and October 2016. To make the results generalizable to a broad population, we wanted to obtain information from people with different backgrounds, educational levels, age, and not just university students. Therefore, participants were recruited via social media, websites^2^,^3^,^4^, mailing lists, as well as posters advertising the study on Campus, cafes and community centers around Brighton. Inclusion in a £25 prize draw or a possibility to earn two study credits for Psychology undergraduate students were offered as an incentive for participation.

### Procedures

After reading study information, volunteers confirmed that they understood all information and then consented to their willingness to take part in the study. After completing the survey, participants were debriefed. The completion of the study took approximately 20 min (based on a pilot study during which participants completed the study uninterrupted).

### Questionnaires

Basic demographics questionnaire was used to determine age, sex, education, smoking habits, and recreational drug use.

Alcohol Use Questionnaire (Townshend and Duka, 2002) provided an estimate of a number of alcohol units consumed a week.

UPPS-P Impulsive Behavior Scale (Whiteside and Lynam, 2001; Cyders and Smith, 2007) is a 59-item self-report measure of five dimensions of impulsivity: negative urgency (NU) – a tendency to act on impulse while experiencing strong negative emotions, (lack of) premeditation (LPrem)– a tendency to act without taking into account the consequences, (lack of) perseverance (LPe) – difficulty completing tasks which may be tedious or difficult, sensation seeking (SS) – a pursue of excitement and novelty, and positive urgency (PU) – a tendency to act on impulse while experiencing strong positive emotions.

Behavioral Inhibition System/Behavioral Activation System (BIS/BAS) Questionnaire (Carver and White, 1994) consists of 20 items organized into two main scales: BIS, which evaluates punishment sensitivity, and BAS which assesses reward sensitivity. BAS is further divided into three subscales: BAS Reward (anticipation or the occurrence of the reward), BAS Drive (the pursuit of desired goals), and BAS Fun Seeking (desire for new rewards and willingness to approach them).

Body Perception Questionnaire (BPQ) Very Short Form (Porges, 1993) consists of 12 items rated on a five-point scale and provides a measure of general awareness of bodily processes (high values indicate high awareness of bodily sensations).

Depression, Anxiety, Stress Scale (DASS) (Henry and Crawford, 2005) consists of three 7-item self-report scales that measure the extent of depression, anxiety, and stress experienced over the last week.

Positive Affect/Negative Affect Scale (Watson et al., 1988) is a 20-item measure of self-reported positive (PA), and NA experienced at the present moment.

### Tasks

Matching Familiar Figures Task (MFFT) (Kagan et al., 1964; Cairns and Cammock, 1978) is a measure of reflection impulsivity. Participants need to identify an image identical to a target one, out of six possible options. The dependent variable is an Impulsivity Score (IS), which reflects quick responses and a high number of errors (high values indicate high reflection impulsivity).

Monetary Choice Questionnaire (Kirby et al., 1999) is a measure of temporal impulsivity. It consists of a list of 27 choices between pairs of smaller immediate rewards (SIR) and larger but delayed rewards (LDR). The dependent variable is the discounting parameter (*k*) calculated for each participant using the formula: *k* = ((LDR-SIR)-1)/delay (log-transformed to reduce skewness). Large *k* values indicate high temporal impulsivity.

Probability Discounting task (Madden et al., 2009) is a measure of risk-taking. It consists of a list of 30 choices between smaller certain rewards and uncertain larger gains. The dependent variable is *h*-parameter, which reflects a degree of probability discounting at the indifference between two outcomes (a point at which the certain and probabilistic rewards are of equivalent subjective value). The *h*-parameter was calculated for each participant using the formula: *h* = (ProbabilisticReward/CertainReward -1)/Odds Agains Winning) (ln-transformed to reduce skewness). Large *h* values indicate discounting of probabilistic rewards (risk aversion).

### Data Analysis

Data analysis was conducted using Statistical Package for Social Sciences (SPSS) version 22. First, QQprincipal component analysis (PCA) with pairwise deletion was conducted to reduce the number of variables for further analysis. PCA was carried out with Varimax rotation with Kaiser Normalization. Next, exploratory correlations between identified components were computed to better characterize their mutual relationship. Finally, multiple regression models were constructed to investigate which components best predict each subtype of impulsive behavior.

## Results

### Participants

603 individuals completed the online questionnaire (132 males; age 18–74 24.39 ± 9.26), of whom 183 were 1st or 2nd-year psychology students who took part in the study in exchange for course credits. Due to such variability in age and a small fraction of older volunteers, we decided to focus on a subset of younger participants (≤45 years old). Therefore, the final sample size was constrained to 574 (121 males; age 18–45, 22.83 ± 6.06). 474 participants were non-smokers.

### Exclusions

The following exclusion criteria were employed: for the MCQ and PD, participants with low response consistency (<75%) were excluded from the analysis (23 and 6 excluded, respectively), as low consistency makes it difficult to establish the discounting parameters reliably. Due to the specific character of the study and limited control over circumstances participants were completing the tasks, for the MFFT, for which response time is important for calculating the dependent variable IS, we excluded participants whose reaction times were outside the range observed in the previous study performed in our lab with a large sample size (*N* = 160) (Caswell et al., 2015) (46 excluded).

### Principle Component Analysis

Eighteen variables were included in the PCA: mean *k* value (log10-transformed to correct issue of non-normality), mean *h* value (ln-transformed), MFFT IS, NU, PU, LPrem, LPe, SS, BIS, BAS Fun, BAS Reward, BAS Drive, BPQ, Depression, Anxiety, Stress, PA, NA.

The total sample size of 574 participants for the 18 items exceeds the suggested minimum ration of five participants per item (Gorsuch, 1983). Chi-square was used to evaluate the fit between the model and the data. Components with eigenvalues > 1 were retained, yielding six components, with the total of 67% of variance explained, which seemed to fit the data well. The Kaiser-Meyer-Olkin measure of sampling adequacy was.757, above the commonly recommended value of.6, and Bartlett’s Test of Sphericity was significant (χ^2^ (153) = 3107.60, *p* < .001), indicating that the null hypothesis that the correlation matrix is an identity matrix can be rejected. Finally, the communalities were all above.4, further confirming that each item shared some common variance with other items. Three items (PA BAS reward, and BPQ) cross-loaded on two factors above.4. Overall, PCA was deemed to be suitable for all 18 items. For details see **Table 1**.

**Table 1:** Component loadings and reliability scores for components identified with the PCA.

	RC 1	RC 2	RC 3	RC 4	RC 5	RC 6
Anxiety	**0.850**	-0.033	0.065	0.057	0.119	-0.069
BAS Drive	0.095	**0.766**	-0.044	-0.037	-0.057	0.079
BAS Fun	-0.039	**0.810**	0.294	-0.009	0.029	-0.002
BAS Reward	-0.067	**0.671**	-0.240	**0.486**	0.031	0.008
BIS	0.165	-0.128	-0.051	**0.868**	-0.027	0.006
BPQ	0.201	0.099	-0.122	0.137	**0.569**	-**0.495**
Depression	**0.804**	-0.095	0.198	0.082	-0.086	0.034
MCQ log *k*	0.056	0.094	0.170	-0.055	**0.614**	0.049
MFFT IS	0.085	0.069	0.053	0.050	0.173	**0.859**
NA	**0.804**	0.069	0.020	-0.084	0.034	0.054
Negative urgency	0.362	0.324	**0.589**	0.321	0.110	0.072
Positive affect	0.092	**0.449**	-**0.499**	-0.357	0.173	0.064
LPer	0.139	-0.219	**0.776**	-0.028	0.028	0.010
Positive urgency	0.282	0.397	**0.624**	-0.085	0.109	0.033
LPrem	0.016	0.184	**0.773**	-0.187	0.037	0.080
PD ln *h*	-0.084	-0.249	-0.046	-0.007	**0.566**	0.177
SS	-0.126	**0.672**	0.136	-0.355	-0.091	-0.086
Stress	**0.864**	0.037	0.103	0.173	0.015	0.011

Cronbach’s alpha	0.864	0.545	0.665	0.337	0.142	0.096

Variance explained [%]	17.30	15.60	13.65	8.13	6.35	5.87


The first component represented items related to the negative emotional state including Depression, Anxiety, Stress and NA. Component 2 included items related to how behaviors are motivated by the pursuit of rewards and excitement as well as positive feelings (namely all three BAS subscales, SS, and PA). Component 3 contained items related to trait impulsivity (PU, NU, LPe, LPrem; all subscales of UPPS-P impulsivity scale but SS), and PA. Component 4 included punishment avoidance trait (BIS) and BAS reward, and factor 5 contained discounting parameters (*k* and *h*) and BPQ. Finally, factor 6 contained BPQ and MFFT IS.

Removal of PA and SS from component 2, resulted in more reliable BAS factor (α = 0.721), therefore, for the further analysis, we chose to use BAS separately from SS and PA. Likewise, deletion of PA from component 3 resulted in higher reliability score (α = 0.751); therefore, the new Impulsive Personality Trait (IPT) component was computed. The components 4, 5, and 6, had low-reliability scores; thus, these items were kept separately.

The complete list of variables used in subsequent analyses together with descriptive statistics is presented in **Table 2**.

**Table 2:** Final variables identified based on PCA, descriptive statistics and gender scores comparisons.

	All			Female			Male			Levene’s test		*t*-test		
	*N*	M	SD	*N*	M	SD	*N*	M	SD	*F*	*p*	*t*	df	*p*
BPQ	574	2.91	0.93	453	2.92	0.91	121	2.89	1.00	3.90	0.049	0.30	177.44	0.761
SS	574	31.99	7.42	453	31.44	7.46	121	34.07	6.94	1.44	0.231	3.50	572	**<0.001**
BIS	574	22.27	3.75	453	22.83	3.55	121	20.20	3.75	0.31	0.578	7.14	572	**<0.001**
BAS	574	38.58	5.66	453	38.56	5.78	121	38.68	5.19	4.03	0.045	0.22	206.60	0.827
PA	574	26.67	9.00	453	26.30	8.87	121	28.06	9.36	0.68	0.409	1.91	572	0.056
Negative Emotions	574	17.36	7.59	453	58.49	32.08	121	54.99	29.10	0.65	0.420	1.09	572	0.278
MCQ log *k*	551	-2.05	0.76	432	-2.11	0.74	119	-1.80	0.77	0.79	0.374	4.00	549	**<0.001**
PD ln *h*	568	0.69	0.99	448	0.71	0.98	120	0.60	1.01	0.00	0.966	1.13	566	0.260
MFFT IS	533	-0.02	1.36	419	0.01	1.34	114	-0.15	1.43	0.15	0.697	1.15	531	0.251
IPT	574	99.72	20.53	453	99.23	20.91	121	101.59	19.04	0.78	0.377	1.12	572	0.262


### Correlations

The correlational analysis was conducted to explore further and better characterize the relationship between items identified via PCA. Since impulsivity-related traits decrease with age (Steinberg et al., 2008) and our sample had a large age-range (18–45), correlations between all the variables and age were computed. PD *h* parameter was positively correlated with age, indicating increased discounting of probabilistic rewards with age (risk-avoidance), *r*(566) = 0.118, *p* = 0.005. Similarly, SS was negatively correlated with age, *r*(572) = -0.142, *p* = 0.001, indicate a decrease in SS with age. MFFT IS score slightly decreased with age, also indicating a decrease in reflection impulsivity with age, *r*(531) = -0.09, *p* = 0.032. IPT was also negatively correlated with age, *r*(572) = -0.113, *p* = 0.007, suggesting a decrease in trait impulsivity with age. Lastly, positive affect was positively correlated with age, *r*(572) = 0.119, *p* = 0.004.

We also wanted to account for possible sex differences in the identified components. Significant differences were found in SS, BIS scores, and temporal impulsivity (**Table 2**); namely, females reported higher punishment avoidance (higher BIS score), but lower SS, than males. Females also discounted delayed rewards less steeply than males (i.e., showed lower temporal impulsivity).

Therefore, partial correlations were computed between all variables used in the further analysis controlling for age and gender (see **Table 3** for details). Bonferroni correction for multiple comparisons was set at *p* ≤ 0.001.

**Table 3:** Pearson partial correlations, controlling for age and gender, between identified variables.

		SS	BIS		BAS		Positive affect		Neg emotions		IPT		MCQ log *k*		PD ln *h*		MFFT IS	
BPQ	*r*	-0.007	0.055		0.07		0.058		**0.179**	**^∗∗∗^**	0.013		0.094	^∗^	0.047		-0.05	
	*p*	0.877	0.193		0.094		0.168		<0.001		0.75		0.027		0.26		0.251	
	*df*	570	570		570		570		570		570		547		564		529	
SS	*r*		-**0.302**	**^∗∗∗^**	**0.494**	**^∗∗∗^**	**0.252**	**^∗∗∗^**	-0.119	^∗∗^	**0.167**	**^∗∗∗^**	-0.007		-0.109	^∗∗^	-0.012	
	*p*		<0.001		<0.001		<0.001		0.004		<0.001		0.865		0.009		0.781	
	*df*		570		570		570		570		570		547		564		529	
BIS	*r*				-0.028		-**0.191**	**^∗∗∗^**	**0.209**	**^∗∗∗^**	-0.003		0.022		0.008		-0.02	
	*p*				0.506		<0.001		<0.001		0.952		0.614		0.856		0.638	
	*df*				570		570		570		570		547		564		529	
BAS	*r*						**0.303**	**^∗∗∗^**	0.018		**0.23**	**^∗∗∗^**	0.079		-**0.136**	**^∗∗∗^**	0.06	
	*p*						<0.001		0.669		<0.001		0.064		0.001		0.169	
	*df*						570		570		570		547		564		529	
Positive affect	*r*								-0.03		-**0.166**	**^∗∗∗^**	0.035		-0.018		0.009	
	*p*								0.479		<0.001		0.411		0.663		0.84	
	*df*								570		570		547		564		529	
Neg emotions	*r*										**0.359**	**^∗∗∗^**	0.104	^∗^	-0.027		0.086	^∗^
	*p*										<0.001		0.015		0.525		0.047	
	*df*										570		547		564		529	
IPT	*r*												**0.183**	**^∗∗∗^**	-0.035		0.134	^∗∗^
	*p*												<0.001		0.401		0.002	
	*df*												547		564		529	
MCQ log *k*	*r*														0.005		0.091	^∗^
	*p*														0.916		0.041	
	*df*														545		508	
PD ln *h*	*r*																0.045	
	*p*																0.307	
	*df*																524	


*^∗^*p* < 0.05, ^∗∗^*p* < 0.01, ^∗∗∗^*p* ≤ 0.001, in bold are presented correlations that survived the correction for multiple comparison (*p* ≤ 0.001). BPQ – Body perception questionnaire score, SS – Sensation Seeking, BIS – Behavioral Inhibition Scale score, BAS – Behavioral Approach Scale score, Neg Emotions – Negative Emotional State (DASS and NA), IPT – Impulsive Personality Trait, MCQ log *k* – Monetary Choice Questionnaire log transformed *k* parameter, PD ln *h* – Probability Discounting ln transformed parameter *h*, MFFT IS – Matching Familiar Figures Task Impulsivity Score.*

### Mood and Impulsivity

Impulsive Personality Trait, as well as BIS, were significantly correlated with PA and the Negative Emotional state indicating that individuals higher on self-reported impulsivity and punishment aversion also reported lower levels of positive affect and higher levels of negative mood state. The reverse was true for SS – increased SS, which was related to higher positive affect and lower negative emotions. Similarly, BAS was positively correlated with PA, suggesting that individuals high in reward sensitivity experience more positive affect. Temporal discounting and MFFT IS were correlated with the Negative Emotional state indicating that increased negative state was related to an increased temporal and reflection impulsivity. However, these correlations did not survive Bonferroni correction for multiple comparisons.

#### The Relationship Between Behavioral and Trait Measures

Monetary Choice Questionnaire and MFFT only correlated with IPT, indicating increased temporal and reflection impulsivity in high-trait impulsivity individuals. PD, on the other hand, correlated with SS and BAS, suggesting that high SS (did not survive the Bonferroni correction) and BAS was related with impulsive decisions in the PD task (choosing the riskier option).

#### The Relationship Between Personality Traits

SS was negatively associated with BIS, indicating that individuals who were high in SS report low punishment avoidance. Instead, SS, BAS, and impulsive personality were all positively inter-correlated.

### Interoceptive Sensibility and Impulsivity

Body Perception Questionnaire was positively correlated with Negative Emotions component indicating that self-reported bodily awareness is related to increased negative mood. Moreover, BPQ was also weakly positively correlated with MCQ, meaning that individuals high on impulsive personality also reported high self-perceived bodily awareness, however, this correlation did not survive Bonferroni correction.

### Regressions

Multiple linear regressions were conducted with performance on the three behavioral tasks as dependent variables. Sex, mean centered age and items identified with the factor analysis served as independent variables.

ANOVA indicated that all three regression models provided a good fit for the data (MCQ log k: *F*(9, 541) = 5.10, *p* < 0.001; PD ln *h*: *F*(9, 558) = 2.91, *p* = 0.002; MFFT IS: *F*(9, 523) = 2.41, *p* = 0.011). Tests to see if the data met the assumption of collinearity indicated that multicollinearity was not a concern (for all the dependent variables: Tolerance > 0.06, 1 < VIF < 1.7).

It was found that trait impulsivity and sex were both significant predictors of the MCQ *k* parameter. Increased delay discounting (higher temporal impulsivity) was predicted by male sex and higher IPT. None of the measures of mood were predictors; however, BPQ approached significance. Age and BAS were significant predictors of *h* parameter, indicating that younger age and higher reward sensitivity were predictive of more risky behavior on the PD. Trait impulsivity turned out to be the only significant predictor of the MFFT IS, suggesting that high trait of impulsive personality is predictive of reflection impulsivity. Details are presented in **Table 4**.

**Table 4:** Results of the multiple regression.

Dependen variable	Predictors	B	SE	Beta	*t*	Sig.	R	R Square
MCQ log *k*	(Constant)	-2.11	0.04		-58.96	<0.001	0.279	0.078
	IPT	0.01	0.00	0.19	3.92	**<0.001**		
	Gender	0.30	0.08	0.17	3.76	**<0.001**		
	BPQ	0.06	0.03	0.08	1.85	0.065		
	Positive Affect	0.01	0.00	0.07	1.46	0.144		
	BAS	0.01	0.01	0.04	0.84	0.402		
	Age	0.00	0.01	0.03	0.69	0.492		
	Neg Emotions	0.00	0.00	0.01	0.27	0.784		
	BIS	0.00	0.01	0.01	0.17	0.869		
	SS	-0.01	0.01	-0.07	-1.38	0.167		

PD ln *h*	(Constant)	0.31	0.02		15.37	<0.001	0.212	0.045
	BAS	-0.01	0.00	-0.12	-2.39	**0.017**		
	Age	0.01	0.00	0.10	2.37	**0.018**		
	BPQ	0.03	0.02	0.06	1.46	0.144		
	Gender	-0.06	0.05	-0.06	-1.35	0.179		
	SS	0.00	0.00	-0.07	-1.31	0.190		
	Neg Emotions	0.00	0.00	-0.05	-1.07	0.284		
	Positive Affect	0.00	0.00	0.03	0.75	0.454		
	IPT	0.00	0.00	0.03	0.58	0.565		
	BIS	0.00	0.01	0.00	-0.07	0.944		

MFFT IS	(Constant)	0.01	0.07		0.15	0.880	0.198	0.039
	IPT	0.01	0.00	0.12	2.37	**0.018**		
	Age	-0.02	0.01	-0.08	-1.79	0.074		
	BPQ	-0.10	0.06	-0.07	-1.53	0.127		
	SS	-0.01	0.01	-0.08	-1.49	0.138		
	BAS	0.02	0.01	0.06	1.21	0.228		
	Neg Emotions	0.00	0.00	0.05	1.08	0.281		
	BIS	-0.02	0.02	-0.05	-0.94	0.348		
	Gender	-0.13	0.15	-0.04	-0.86	0.391		
	Positive Affect	0.00	0.01	0.03	0.53	0.594		


*BPQ – Body perception questionnaire score, SS – Sensation Seeking, BIS – Behavioral Inhibition Scale score, BAS – Behavioral Approach Scale score, Neg Emotions – Negative Emotional State (DASS and NA), IPT – Impulsive Personality Trait, MCQ log *k* – Monetary Choice Questionnaire log transformed *k* parameter, PD ln *h* – Probability Discounting ln transformed parameter *h*, MFFT IS – Matching Familiar Figures Task Impulsivity Score.*

## Discussion

The current study investigated the role of personality traits (impulsive tendencies, reward sensitivity, punishment avoidance, and interoceptive sensibility) and emotional states as potential modulators of distinct subtypes of impulsive and risky behaviors. In accordance with our hypotheses, we first confirmed that trait impulsivity (IPT; positive and NU and lack of premeditation and perseverance components of the UPPS-P scale) predicted temporal and reflection impulsivity. Moreover, reward sensitivity (BAS) best predicted risk-taking in a PD. However, contrary to our initial predictions, affective state did not predict any behavioral dimensions and no link was found between subjective interoception (interoceptive sensibility) and risk-taking.

We hypothesized that negative emotional state would relate to decreased self-control and therefore more impulsive behavior. Although mood state was not a predictor of any of the behavioral tasks, we found correlational evidence providing tentative support for our hypothesis. Specifically, negative emotional state was related to both more short-sighted monetary decisions (increased temporal impulsivity) and more rushed decisions in the MFFT (increased reflection impulsivity). Although these relationships were weak, they nevertheless added to evidence form earlier studies which have suggested that the experience of emotional distress, drives people to treat themselves to immediate pleasures, such as indulgent foods over healthy options, as a means of regulating one’s mood (Moore et al., 1976; Tice et al., 2001; Lerner et al., 2013; Gardner et al., 2014). Experience of emotional distress is also considered a major trigger in substance use relapse. For example, stressful events increase the urge to drink alcohol and chances of relapse in treated alcoholics (Sinha et al., 2009; Sinha, 2012). Increasingly, research also suggests that people drink alcohol to enhance positive or manage negative emotional state, and reduce tension (Conger, 1956; Cooper et al., 1995; Zack et al., 2002). Together, these findings support the importance of emotional state in impulsive choice and suggest that negative emotions bias behavior toward rushed and more near-sighted decisions, which can further lead to detrimental consequences both regarding finance (e.g., self-indulgence to improve one’s mood instead of saving) and health (obesity, the risk of cardiovascular disorders, substance misuse).

A relationship was also observed between emotional state and trait measures: High levels of positive affect were associated with high levels of SS and reward impulsivity (SS and BAS) and low levels of both BIS and impulsive traits (IPT). The reverse was true for high levels of negative emotions. The fact that self-reported trait measures were related to state mood-measures merits comment since they are usually considered to be stable personality traits, unaffected by changes in mood (Weafer et al., 2013). The positive association between self-reported impulsivity and negative emotions corroborates with findings from clinical populations indicating increased impulsive tendencies in depressed individuals (Peluso et al., 2007; Tomko et al., 2015). Moreover, similarly to previous research (Sperry et al., 2016), higher SS ratings were associated with higher positive affect.

However, since these are correlational measures, causality cannot be assumed. Nevertheless, it is plausible that while experiencing negative emotions, individuals may recall events when they behaved impulsively (memory bias) and be primed to behave the same way. Alternatively, engaging in impulsive actions may serve as a way of regulating one’s mood (Tice et al., 2001). Thus it seems that emotional state is a consideration when assessing trait impulsivity.

It is noteworthy that the IPT (IPT; as identified here) was related to negative emotions, whereas levels of SS were associated with positive affect. This dissociation between impulsive and risk-taking traits was further supported by component loadings within the PCA, which separated SS from the remaining UPPS-P subscales. Indeed, although SS is encompassed within some constructs of impulsivity (Zuckerman, 1984; Whiteside and Lynam, 2001), other research suggests a differentiation between these two concepts (Magid et al., 2007). Our findings also show that SS is distinct from trait impulsivity.

Delay discounting and reflection impulsivity were both predicted by the self-reported impulsivity (IPT), while risk-taking (probability discounting) was explained solely by BAS. Indeed, although early research suggests that delay and probability discounting are both facets of impulsive choice, sharing underlying processes (e.g., Rachlin, 1990; Mazur, 1993; Richards et al., 1999), more recent work argues that these two concepts are distinct from each other (Holt et al., 2003; Madden et al., 2009; Shead and Hodgins, 2009). Our findings agree with the latter, suggesting that delay and probability discounting reflect distinct aspects of decision-making, indexing delayed gratification and risk-taking/reward sensitivity, respectively.

In agreement with an earlier report (Silverman, 2003), we observed that males showed significantly more delay discounting than females. The reason why gender may play such a role, what the mechanisms and potential consequences are, should be a subject of the future research.

Impulsive personality traits, which include facets of emotional impulsivity, predicted performance on the delay discounting task, supporting our hypothesis. It is worth noting that in both delay and probability discounting, our models explained only a small fraction of the variance, which suggests that other factors are contributing to discounting which are yet to be identified.

The MFFT task has been widely used to study reflection impulsivity in children and other target populations (Kagan, 1965; Verdejo-García et al., 2008; Carretero-Dios et al., 2009). However, it has been heavily criticized as a measure of behavioral impulsivity (e.g., Block et al., 1974) and suggested to be more related to cognitive performance more generally rather than behavioral impulsivity (Block et al., 1986; Perales et al., 2009). Our results indicate that IPT is the best predictor of performance on the MFFT task, also supporting the classification of MFFT performance as a measure of reflection impulsivity (Caswell et al., 2015).

In contrast to our expectations, no relationship was found between subjective interoceptive sensibility (BPQ) and probability discounting. This is distinct from previous research which reported the relationship between risk-taking or disadvantageous decision-making and individual differences in interoception (Werner et al., 2009; Kandasamy et al., 2016). These discrepancies may be due to methodological aspects of the measures employed. In the current study, we used a PD, which is an explicit measure of risk-taking. Using a more implicit measure of risk-taking, e.g., a gambling task, alongside a dimensional approach to quantifying (subjective objective and metacognitive) interoceptive abilities (Garfinkel et al., 2015) could provide much finer grained insight into how interception relates to impulsivity, extending previous findings. Instead, we found a trend for bodily awareness to predict temporal discounting, indicating that heightened subjective sensitivity to bodily sensations (i.e., higher interoceptive sensibility, often characteristic of more anxious individuals) may result in increased temporal impulsivity. Similarly, the observed relationship between BPQ and negative emotions is also consistent with the association between interoception and anxiety (e.g., Pollatos et al., 2009; Dunn et al., 2010; Stevens et al., 2011; Garfinkel et al., 2015).

## Limitations

Some study limitations merit comment. Firstly, this study relied on survey data obtained via an online questionnaire. There was consequently little experimental control over the circumstances in which participants completed the study, which should be taken into account. Future research may benefit from more controlled environments, e.g., as a typical lab-based study, to validate these findings. Secondly, despite recruiting participants online, our sample consisted mainly of female participants and a very small proportion of older adults. In the future, a more gender-balanced sample also including elderly should be studied to confirm these findings.

## Conclusion

Our results indicate that IPTs predict temporal and reflection impulsivity, while reward sensitivity predicts risk-taking behavior (probability discounting). This separation between measures of impulsivity and risk-taking suggests that the two concepts are distinct. The dissociation between measures of impulsivity and risk-taking was further highlighted by their relationship to the current emotional state: While increased negative emotions were predictably associated with increased impulsivity, increased positive affect was associated with increased measures of risk-taking. This interesting finding has important consequences for research since it suggests that the same person may show different levels of trait impulsivity in a positive (less impulsive) than a negative (more impulsive) mood state. Thus, future research into trait impulsivity should attend to concurrent mood states of participants. Marginal findings of the present study also motivate areas of further research: The fact that negative emotions were related to increased temporal impulsivity may indicate at least partly why people in a positive mood are likely to make commitments, such as keeping to a diet or exercising regularly – that is when they can oversee long-term goals over immediate ones. Consequently, in a negative emotional state, perception shifts toward immediate gratification (e.g., comfort food, watching television series instead of going to the gym). Moreover, our findings with the BPQ link subjective body awareness to temporal impulsivity suggesting the need for in-depth understanding of the relationship between interoceptive ability and decision-making.

## Data Availability Statement

The data that support the findings of this study are available from the corresponding author upon reasonable request.

## Ethics Statement

This study was carried out in accordance with the recommendations of the University of Sussex Ethical board. The protocol was approved by the Sciences & Technology Cross-Schools Research Ethics Committee. All subjects gave written informed consent in accordance with the Declaration of Helsinki.

## Author Contributions

AH collected and analyzed data and wrote the initial manuscript. AH and TD interpreted the results. TD provided a guidance throughout. All authors contributed to the experimental design and the final version of the manuscript.

## Conflict of Interest Statement

The authors declare that the research was conducted in the absence of any commercial or financial relationships that could be construed as a potential conflict of interest.

## Footnotes

1 https://www.qualtrics.com/

2 https://www.reddit.com/

3 https://www.craigslist.org/

4 https://www.callforparticipants.com/
